# Global Burden of Nutritional Deficiencies among Children under 5 Years of Age from 2010 to 2019

**DOI:** 10.3390/nu14132685

**Published:** 2022-06-28

**Authors:** Tingting Yue, Quanquan Zhang, Guangdi Li, Hong Qin

**Affiliations:** 1 Hunan Provincial Key Laboratory of Clinical Epidemiology, Xiangya School of Public Health, Central South University, Changsha 410078, China; yuetingting@csu.edu.cn (T.Y.); zquanquan2020@163.com (Q.Z.); 2 Department of Nutrition Science and Food Hygiene, Xiangya School of Public Health, Central South University, 110 Xiangya Road, Changsha 410078, China

**Keywords:** children, global burden, nutritional deficiencies, socio-demographic indexes, human development index

## Abstract

Under-five years of age is a critical period for children’s growth and development. Nutritional deficiency during this period is associated with wasting, underweight and stunting. We aimed to conduct an epidemiological study using data derived from the GBD2019 to found the global distribution and changing trends of nutritional deficiencies among children under 5 years old, as well as the correlation between social development status and nutritional deficiencies. Nutritional deficiencies in children under 5 years has been substantially improved in the past decade; however, the progress has been unevenly distributed globally. The incidence and DALY rate decreased with the increase of socio-demographic index. In 2019, the incidence (51,872.0 per 100,000) was highest in Central Sub-Saharan Africa and the DALY rate (5597.1 per 100,000) was the highest in Western Sub-Saharan Africa. Among five subcategories of nutritional deficiencies in children under 5 years, vitamin A deficiency accounted for the largest proportion of incident cases (100,511,850, 62.1% in 2019), while the proportion of DALYs caused by protein–energy malnutrition was the highest (9,925,276, 62.0%). Nutritional deficiency in some countries remains worrisome, for whom policies guarantees and sustained efforts to control nutritional deficiencies are urgently needed.

## 1. Introduction

Nutritional deficiency is an important factor that can affect health and increase the risk of acute infectious disease or death [1,2,3]. The under-five is a critical period for children’s growth and development and a “vulnerable period” to a variety of factors, including nutritional deficiencies [4]. Under-nutrition during this period is related to wasting, underweight and stunting, thus, damaged child’s own health [5,6,7]. There is also evidence that undernutrition in early childhood can affect cognition, language and social emotions, hinder later development and have a negative impact on human capital and economic growth [8,9,10].

In 2019, 161,847,936 children under five years of age suffer from nutritional deficiencies globally [11]. Nutritional deficiency in children is a public health problem that cannot be ignored and it is necessary to grasp its status quo to propose preventive measures based on this. Nutritional deficiencies mainly include micronutrient (vitamin and minerals) deficiencies and protein–energy malnutrition [12]. Vitamin A deficiency and iron deficiency are the main forms of micronutrients deficiency in children [13,14,15,16]. Vitamin A deficiency is an important cause of night blindness, exophthalmia and infectious diseases [17,18].

It is reported that an estimated 250,000–500,000 children who are vitamin A-deficient become blind every year, and half of them die within 12 months of losing their sight [7,19]. Iron deficiency is the main cause of anemia, with 41.7% of children worldwide having iron deficiency. According to WHO estimates, 41.7% of children worldwide have iron deficiency, and iron deficiency is the main cause of anemia [20]. In fact, iron deficiency can lead to negative consequences on attention, learning and school performance later in life before anemia sets in [21,22]. Protein–energy malnutrition can cause stunting, infections, cognitive and behavioral disorders and even death [10]. In addition to impairing health, nutritional deficiencies can cause social problems, such as less schooling, lower academic performance and reduced economic productivity [23], thereby, imposing a heavy burden on society.

The United Nations has made end hunger (achieve food security, improve nutrition and promote sustainable agriculture) one of its sustainable development goals by 2030 and nutrition has become a strategic issue [24]. By studying the global burden of nutritional deficiencies and its different subcategories, the major subcategories that cause burden of disease in different regions can be identified, and more targeted measures can be taken. However, there are no such reports in children under 5 years of age [12,25,26].

The Global Burden of Disease Study (GBD) study is the largest and most comprehensive global observational epidemiological survey to date, covering the disease burden of more than 200 countries from 1990 to 2019 [27]. Compared with studies using a single country, GBD data can overcome the impact of research heterogeneity and make up for the lack of relevant data in some countries. Here, we analyzed the data extracted from the GBD2019 to study the disease burden and changing trends of nutritional deficiencies among children under 5 years of age from 2010 to 2019, in the hope that we can provide directions and evidence for fighting nutritional deficiencies in children and contribute to the improvement of nutritional deficiencies in children under 5 years of age.

## 2. Materials and Methods

### 2.1. Data Acquisition

The data used in this study were retrieved from the GBD2019 database. The annual incident cases, incidence, number and rate of DALYs of children under 5 years of age with nutritional deficiencies by region, country and main subcategories of nutritional deficiencies (vitamin A deficiency, dietary iron deficiency, iodine deficiency, protein–energy malnutrition and other nutritional deficiencies) from 2010 to 2019 were extracted from the database (http://ghdx.healthdata.org/gbd-results-tool, accessed on 27 March 2022). Nutritional deficiencies were identified according to the 10th revision International Classification of Diseases and Injuries (ICD-10), including protein energy malnutrition (coded as E40–E46.9 and E64.0), iodine deficiency (coded as E00–E02), dietary iron deficiency (D50–50.9), vitamin A (E50–E50.9 and E64.1) and other nutritional deficiencies (coded as D51–D53.9, E51–61.9, E63–E64 and E64.2–E64.9).

The socio-development index (SDI), developed by GDB researchers, is a comprehensive indicator of social development closely related to health status and outcomes. It is the geometric mean of 0 (worst) to 1 (best) indices of total fertility rate under the age of 25, mean education for those ages 15 and older and lag distributed income per capita [28]. 204 countries and territories are divided into five regions, including low SDI, low-middle SDI, middle SDI, high-middle SDI and high SDI, according to the social demographic index (SDI). The world is separated into 21 regions according to geographic locations, such as East Asia and Central Europe. SDI was used in this study to determine the relationship of socioeconomic development status with incidence and DALY rate for nutritional deficiencies. The SDI data of 204 countries and territories, five regions divided by SDI and 21 geography regions were collected from GBD database (https://ghdx.healthdata.org/data-type/estimate, accessed on 27 March 2022).

The Human Development Index (HDI) is the geometric mean of normalized indices for three dimensions, including a long and healthy life, being knowledgeable and having a decent standard of living. To explore the relationship of HDI and estimated annual change percentage (EAPC) of incidence and DALY rate of nutritional deficiencies, we collected HDI2019 data covering 189 counties from the Human Development Reports of the United Nations Development Program (https://hdr.undp.org/en/composite/HDI, accessed on 27 March 2022).

### 2.2. Statistical Analysis

We used the incidence rate, DALY rate and estimated the annual percentage change (EAPC) to quantify the global burden of nutritional deficiencies of children under 5 years of old. The incidence rate and DALY rate were provided by the GBD 2019 database. The methodology used to estimate the rates and their 95% uncertainty interval (95%UI) was discussed previously [29]. EAPC is a summary and widely used measure of the trend of rate over a specific time interval and is calculated with linear regression models using the following formula [30]: y = α + βx + ϵ, in which y = ln (rate) and x = calendar year. The EAPC was defined as 100 × (exp (β) − 1), and its 95%UI was determined by the regression model.

The changes in the number of incident cases and DALY cases were calculated by comparing the data for the year 2019 to the data for the year 2010. To comprehensively analyze the incidence and DALY rates of nutritional deficiencies in children and the change of them across 204 countries and territories, a hierarchical cluster analysis was performed according to incidence and DALY rates as well as the EAPC of them. The countries and territories with similar incidence and DALY rates or with similar annual changes will be categorized into one cluster.

The correlation between EAPCs and incidence rate in 1990 as well as HDI in 2019 in different countries was evaluated using Pearson correlation analysis to define the potential factors affecting EAPCs. Pearson correlation analysis was also used to explore the correlation between SDI and incidence rate or DALY rate. Locally weighted regression (LOESS)-smoothed method was used to fit the curves of incidence or DALY rate with SDI, as well as the curves of EAPC with HDI or incidence. Statistical analysis was performed using R (version 4.1.0). *p* value < 0.05 was considered statistically significant. All rates are reported per 100,000 person-years.

## 3. Results

### 3.1. Global Burden of Nutritional Deficiencies

#### 3.1.1. Incidence of Nutritional Deficiencies

**Figure 1:**
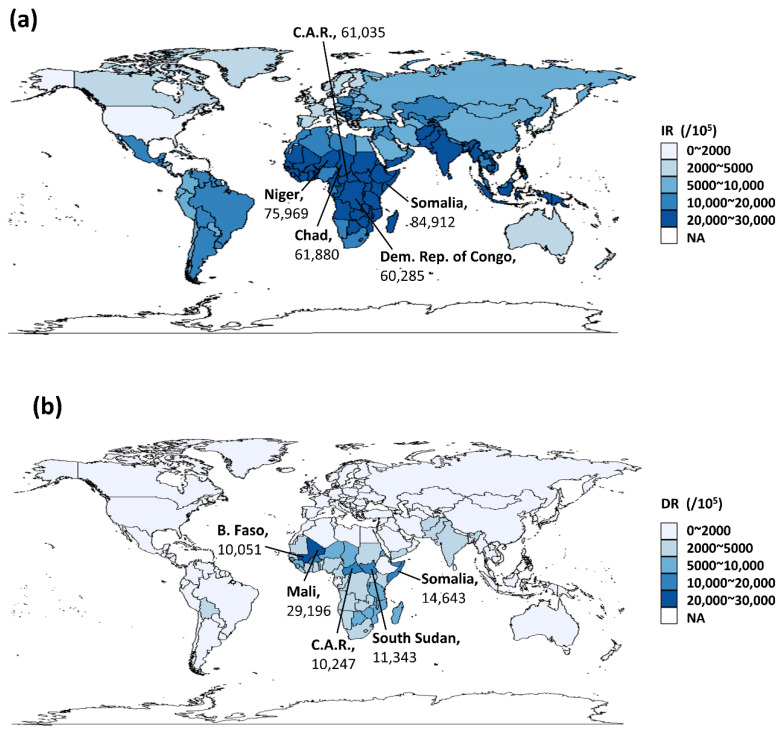
Global burden of nutritional deficiencies in children under 5 years of age. (**a**) Incidence rate of nutritional deficiencies in children under 5 years of age in 2019; (**b**) DALYS rate of nutritional deficiencies in children under 5 years of age in 2019. (IR: Incidence rate; DR: DALYs rate; EAPC: Estimate annual percentage change; C.A.R.: Central African Republic; Dem. Rep. of Congo: Democratic Republic of the Congo; B. Faso: Burkina Faso; and Syr.: Syrian Arab Republic).

The incidence of nutritional deficiencies in children under 5 years of age varied across 204 countries and territories. The highest incidence of nutritional deficiencies in 2019 was observed in Somalia (84,912 per 100,000 population), followed by Niger, Chad, Central African Republic and Democratic Republic of the Congo (Figure 1a). From a global perspective, the overall incidence of nutritional deficiencies in children decreased from 2010 to 2019, with almost all the EAPC of incidence less than 0 (Figure S2a). For the 21 GBD regions, the incidence of 51,872.0 per 100,000 in 2019 made Central Sub-Saharan Africa the highest. Among the five SDI regions, the rate decreased with the increase of SDI, and the lowest was in the high SDI regions (3466.7 per 100,000 in 2019).

**Figure 2:**
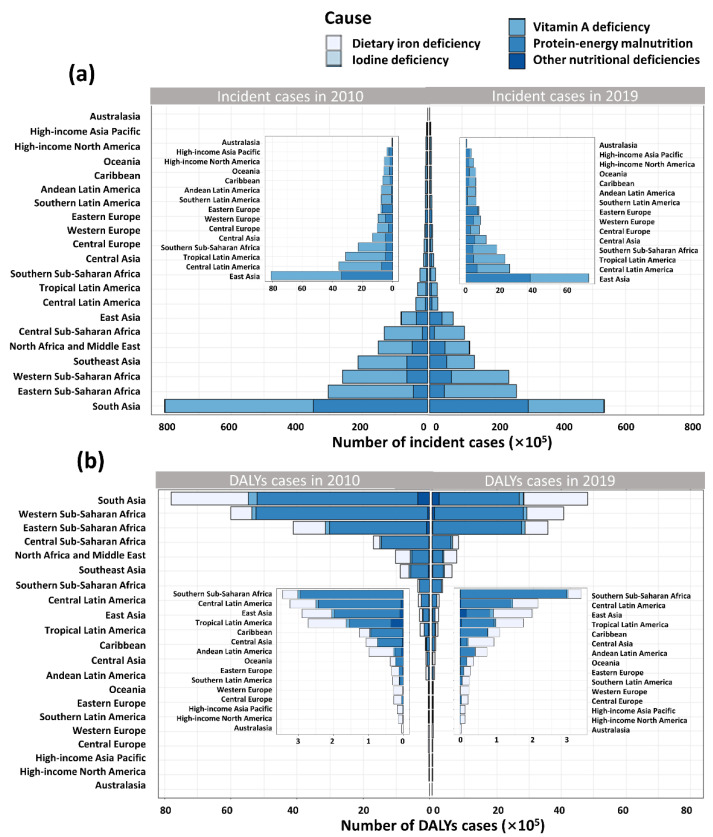
Nutritional deficiencies in children under 5 years of age caused by five different subcategories of nutritional deficiencies in 21 GBD regions. (**a**) Number of incident cases of five subcategories of nutritional deficiencies. (**b**) Number of DALYs cases of five subcategories of nutritional deficiencies.

**Figure 3:**
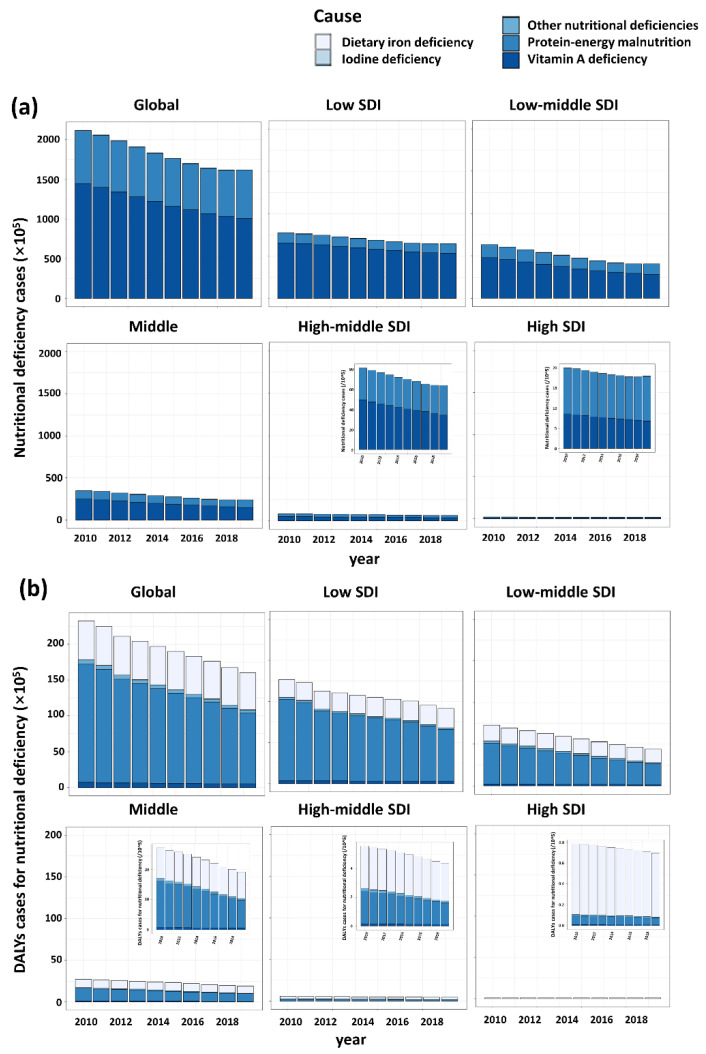
Nutritional deficiencies in children under 5 years of age caused by five different subcategories of nutritional deficiencies in five SDI regions. (**a**) Number of incident cases of five subcategories of nutritional deficiencies. (**b**) Number of DALYs cases of five subcategories of nutritional deficiencies.

Totally, there were 161,847,936 nutritional deficiencies cases of children around the world in 2019. The incident cases of nutritional deficiencies in children decreased 23.5% from 211,593,000 in 2010 to 161,847,935 in 2019 globally. The most pronounced decrease and increase was observed in the Syrian Arab Republic (59.3% decrease) and Somalia (31.2% increase), respectively (Figure S1a). For the 21 GBD regions, the highest number of incident cases were recorded in South Asia, whereas the lowest was in Australasia (Figure 2A). From 2010 to 2019, the number of incident cases in high SDI region remained stable, while the number declined over time in the other four regions (Figure 3a).

#### 3.1.2. DALYs of Nutritional Deficiencies

The DALY rate of Mali ranked first in 2019 (29,196 per 100,000), followed by Somalia, South Sudan, Central African Republic and Burkina Faso (>100,000 per 100,000) (Figure 1b). From 2010 to 2019, the DALY rate showed a similar downward trend as to incidence; however, there were still some countries with an EAPC greater than 0 for DALYs, among which Mali having the highest EAPC of 4.95% (2.76–7.19%) (Figure S2b). The absolute value of EAPC in High-income North America, Australasia, Western Europe, Western Sub-Saharan Africa, High-income Asia Pacific and Southern Sub-Saharan Africa was around 1, indicating that the DALY rate in these regions remained stable (Table 1). For five SDI regions, the highest DALY rate was the in the low SDI regions (5357 per 100,000 in 2019). In the contrast, the lowest was in high SDI regions (132.6 per 100,000 in 2019).

A total of 16,004,379 DALY cases of nutritional deficiencies in children were observed in 2019 (Table 1). Across 204 countries and territories, only a few countries experiencing an increase in number of DALYs overall, among which Mali showed the largest increase (66.7% increase) (Figure S1b). In 2019, the number of DALY cases in South Asia was also the highest, with 4,804,447 cases, down 38.4% compared to 2010. In the contrast, Australasia has the lowest number of DALYs cases (4120 in 2019) (Figure 2B). Among five SDI regions, although the number of DALY cases declined over time, the number still ranked first in the low SDI regions (Figure 3).

### 3.2. Main Subcategories of Nutritional Deficiencies

#### 3.2.1. Incidence of Main Subcategories of Nutritional Deficiencies

Among the five subcategories of malnutrition in children under 5 years of age, vitamin A deficiency accounted for the largest proportion (100,511,850, 62.1% in 2019), although it has significantly decreased (30.5% decrease) compared with 2010, followed by protein energy malnutrition (61,023,138, 37.7% in 2019) and iodine deficiency (312,948, 0.2% in 2019). There were no new cases of dietary iron deficiency and other malnutrition after 2010. The incidence rates of vitamin A deficiency and iodine deficiency were highest in the low SDI region (31,257.5 and 97.1 per 100,000, respectively), while that of protein–energy malnutrition was observed as the highest in the low-middle SDI region, followed by the low SDI region.

As the SDI increased, the proportion of vitamin A deficiency declined in each region, and thus the proportion of protein–energy malnutrition increasing relatively (Figure S3). However, the absolute number of incident cases of three subcategories of malnutrition were most in the low SDI region and decreased with the increase of SDI (Figure 3a). For 21 GBD regions, the proportion of vitamin A deficiencies of total number of nutritional deficiencies in Southern Latin America has ranked first at 83.6%.

Australasia has the largest proportion of protein–energy malnutrition at 93.7% (Figure S3). However, the number of incident cases of Australasia was lowest in the three subcategories of malnutrition, whereas that of South Asia ranked first among 21 GBD regions. As for the incidence, Central Sub-Saharan Africa was the region on the top in Vitamin A deficiency (44,156.8 per 100,000) and Iodine deficiency (455.6 per 100,000). South Asia ranked first in protein–energy malnutrition (18,346 per 100,000) (Table S1).

#### 3.2.2. DALYs of Main Subcategories of Nutritional Deficiencies

The proportion of DALYs caused by protein–energy malnutrition was the highest (9,925,276, 62.0%), followed by dietary iron deficiency (51,798,985, 32.4%) and vitamin A deficiency (505,633, 3.2%). Globally, except for when caused by dietary iron deficiency being stable, the DALY rate of other subcategories of malnutrition showed a decreasing trend. The change of DALY rate caused by protein energy malnutrition was the largest (EAPC = −5.6 [−5.8 to −5.4]) (Table 1).

The rate attributable to protein–energy malnutrition was the highest in Eastern Sub-Saharan Africa (4289.1 per 100,00 in 2019), while that of dietary iron deficiency was the highest in Western Sub-Saharan Africa (1567.0 per 100,00 in 2019). For the five SDI regions, the DALY rates caused by five subcategories of nutritional deficiency were the highest in the low SDI region, among which protein–energy malnutrition was the highest (3703.0 per 100,000), followed by dietary iron deficiency (1377.9 per 100,000) (Table S2).

The number of DALYs due to protein–energy malnutrition is the highest in all the 21 regions, and Eastern Sub-Saharan Africa is the most affected (2,750,984 in 2019), followed by dietary iron deficiency, with South Asia the most (1,969,363 in 2019). In the contrast, the lowest DALY cases cause by all the five subcategories malnutrition were observed in Australasia, and DALY cases caused by dietary iron deficiency were the most (3880 in 2019) (Figure 2B). Among the five SDI regions, similarly, the number of DALYs of five subcategories of nutritional deficiency were highest in the low SDI region and lowest in high SDI region (Figure 3b).

### 3.3. Correlation between SDI and Incidence or DALY Rate

**Figure 4:**
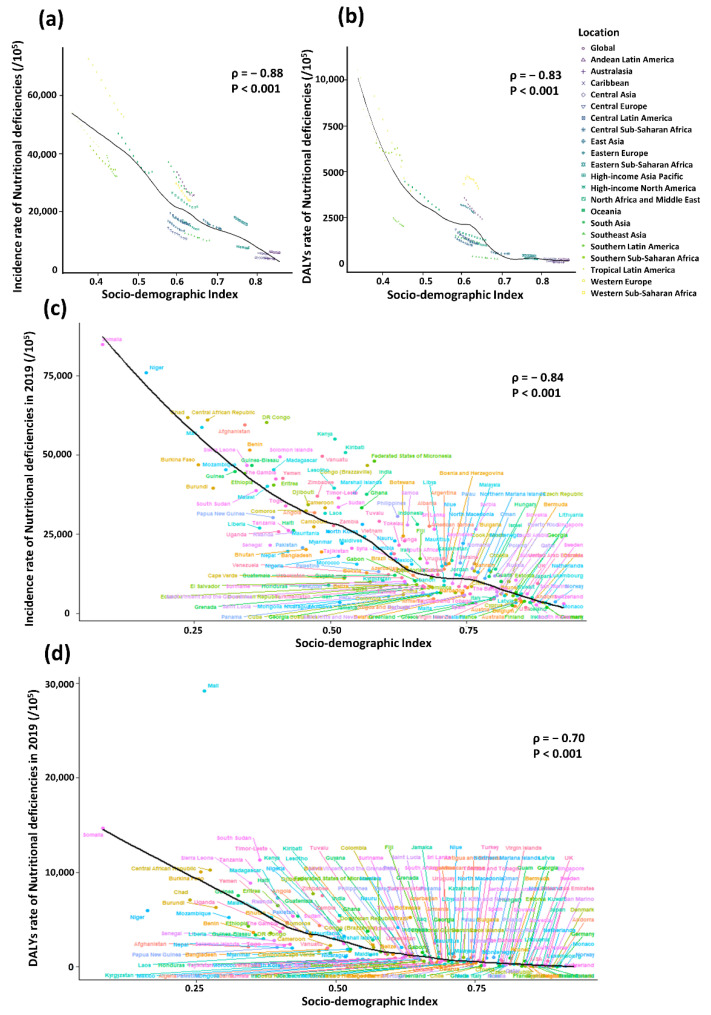
The correlation between socio-demographic index and incidence or DALY rate of nutritional deficiencies, 2010–2019. The correlation between socio-demographic index and incidence (**a**) or DALY rate (**b**) of nutritional deficiencies among 21 GBD regions and five SDI regions. The correlation between socio-demographic index and incidence (**c**) or DALY rate and (**d**) of nutritional deficiencies among 204 countries and territories.

As shown in Figure 4, a significant association was detected between SDI and incidence of nutritional deficiencies, as well as SDI and DALY rates. The expected values based on SDI and the incidence or DALY rates in all regions are shown as a black line. Except for some regions with high SDI, such as Caribbean and Central Asia, where the incidences remained stable. However, there were regions in which the observed incidences were much higher than expected level, such as Tropical Latin America, while the incidences of some regions were lower than expected, such as Central Europe (Figure 4a).

In the correlation analysis between 204 countries and territories and SDI in 2019, incidence was negatively correlated with SDI in the majority countries, except few countries significantly higher or lower than expected. For example, Kenya and Kiribati are far above the expected level whereas Mongolia and Nepal are below the expected level (Figure 4c).

Similarly, the DALY rates of many of the regions decreased with the increase of SDI, except some regions fluctuating or staying stable. For instance, with the increasing of SDI, the DALY rate of Southern Sub-Saharan Africa with low SDI declined at first, then increased briefly and then continued to decrease, while the rate of Western Sub-Saharan Africa with middle SDI rose at first and then declined. The rate of East Asia with high-middle SDI and Andean Latin America with high SDI, however, stay stable during the study period.

Furthermore, almost all the observed DALY rates of regions with high SDI closely followed expected trends from 2010 to 2019, such as Australasia, Caribbean, Andean Latin America and Andean Latin America. However, some regions staying well below expected levels throughout the study period, such as Southern Latin America, while the others above the expected levels, such as Western Europe (Figure 4b). In 2019, similar to the correlation between incidence and SDI, a significant negative association (ρ = −0.70, *p* < 0.001) was found between DALY rates and SDI at the national level, with some exceptions (Figure 4d).

### 3.4. The Influential Factors for EAPC

**Figure 5:**
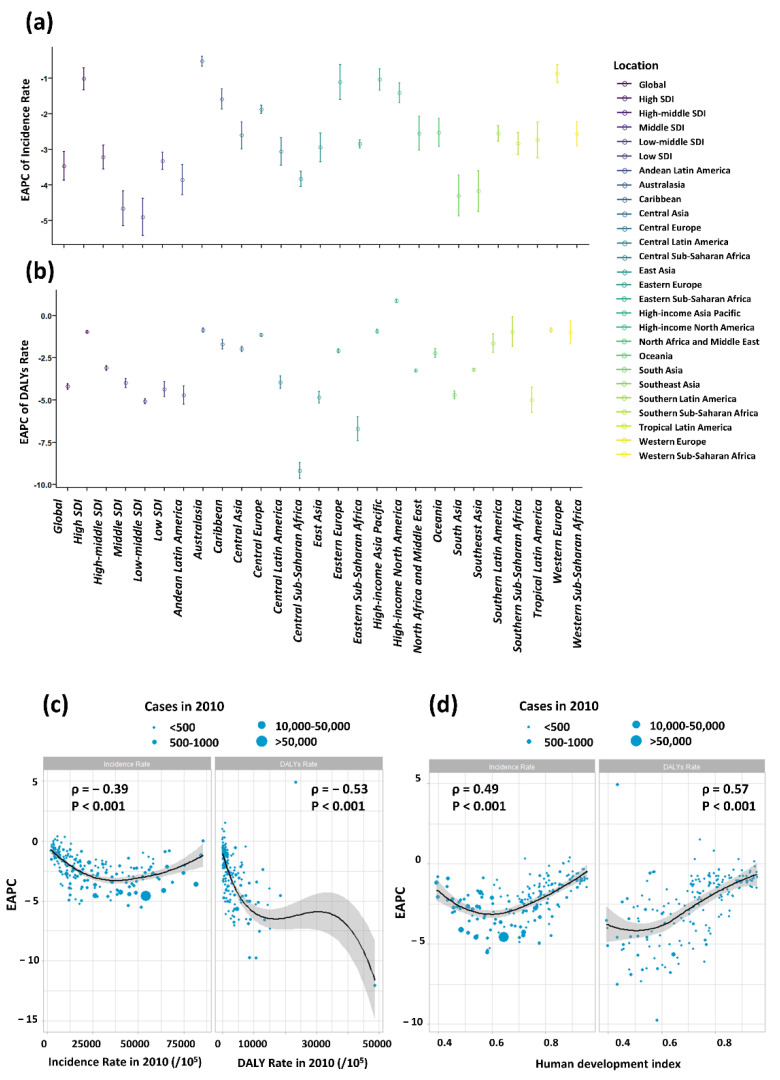
The EAPCs of nutritional deficiencies at global, regional and national level. (**a**) The EAPC of nutritional deficiencies incidence from 2010 to 2019, both sexes, by region and by causes; (**b**) The EAPC of nutritional deficiencies DALYs rate from 2010 to 2019, both sexes, by region. The correlation between EAPC and (**c**) of nutritional deficiencies and DALYs rate in 1990 and (**d**) HDI in 2019. The circles represent countries that were available on HDI data. The size of circle is increased with the cases of nutritional deficiencies cases. The ρ indices and *p* values presented in (**c**,**d**) were derived from Pearson correlation analysis.

Among the five SDI regions, the absolute value of EAPCs of incidence increased with the decline of SDI, except the low SDI region (−3.33 [−3.09 to −3.57]), whose absolute value of EAPC even less than that of middle SDI (−4.66 [−4.17 to −5.15]). For the 21 GBD regions, the absolute value of EAPCs in South Asia (−4.30 [−3.73 to −4.87]) ranked the first, followed by Southeast Asia (−4.18 [−3.60 to −4.75]) and Andean Latin America (−3.85 [−3.42 to −4.28]) (Figure 5a). Similar patterns were observed for EAPCs of DALY rate (Figure 5b).

There was an inverse association between the EAPC of incidence and the incidence in 2010 (ρ = −0.39, *p* < 0.001), so as the EAPC of DALY rate and DALY rate in 2010 (ρ = −0.53, *p* < 0.001) (Figure 5c). The higher the incidence in 2010 was, the lower the EAPC was. Given that the EAPCs were below 0, the country having higher incidence in 2010 experienced a more rapid decrease in the incidence of nutritional deficiencies in children. However, a significant positive relation was detected between EAPCs and HDI in 2019, indicating a slower decrease in incidence in high SDI region. The same correlation exists between DALY rate and HDI (ρ = 0.60, *p* < 0.001) (Figure 5d).

## 4. Discussion

The health of children is related to the development of society. The under-five mortality is one of the important indicators to measure societal wellbeing, and nutrition-related factors account for about 45% of its causes [24,31]. Therefore, improving the nutritional status of children under 5 years of age is an important way to promote their healthy growth. Nutritional deficiencies is one of the critical nutritional problems. Therefore, we systematically assessed the burden of nutritional deficiencies in children under 5 years of age in different SDI regions, territories and countries from 2010 to 2019.

Overall, both incidence (from 32,498.9 to 24,417.2 per 100,000) and DALY rates (from 3574.9 to 2414.5 per 100,000) of nutritional deficiencies in children under 5 years of age declined. The cluster analysis results (Figures S4–S6) showed that incidence and DALY rates declined significantly in 50 (24.5%) countries and the two declined slightly or remained stable in 116 (56.7%) countries. The incidence declined significantly but DALY rates declined slightly in 36 (17.6%) countries. There are numerous factors associated with nutritional deficiencies in children, for example, agriculture and the food environment, antenatal care and socioeconomic factors [32].

The reason why nutritional deficiencies decline globally can be summarized as following: (i) With the promotion of nutrition-sensitive agriculture (A new concept that differs from the traditional focus on grains and oilseeds. It advocates the promotion of food diversification by increasing the variety of crops, aquaculture, livestock and dairy programs), increased diversity of nutritious food production and household access to nutritious food have improved the food environment and thus the quality of mothers’ and young children’s diets [33]. (ii) Antenatal care helps detect and resolve various health problems in a timely manner, and its coverage has increased, providing greater protection for the nutritional status of women and children [34].

(iii) In recent years, the global social economy has continued to develop. Economic growth is associated with access to more nutritious foods and better medical treatments, which leads to improved nutritional status. The relationship between socioeconomics and many diseases have been proven [2,35]. In this study, we also analyzed the relationship between social development-related indicator SDI and nutritional deficiencies and the inverse relationship was found between them.

However, against the background of the global decline in diseases burden of nutritional deficiencies among children under 5 years of age, the burden was still high and worrying in some countries or regions. The highest number of incident cases and DALYs was found in South Asia, which may be attributable to its larger population and lower social economic level. Compared to the number of incident cases and DALYs, incidence and DALY rate were less affected by population size. Both the rates remained high in some African countries (e.g., Somalia, Niger, Mali and Chad), which suggests that nutritional deficiencies in these regions are of greater concern.

The proportions of main nutritional deficiencies subcategories differed across SDI regions, and the proportion of incident cases in protein–energy malnutrition and proportion of DALYs in iron deficiency increased with the increase of SDI. However, in the low-SDI region, vitamin A deficiency accounts for a high percentage of incident cases and DALYs. The number of protein–energy malnutrition decreased in high-SDI region; however, the relative increase of the proportion in protein–energy malnutrition was due to a greater decrease in cases of other nutrients deficiency.

With regard to iron deficiency, the causes in low- and middle-income countries differ from those in high-income countries, where the former is caused by inadequate iron intake or parasitic infections and the latter by inadequate iron absorption and chronic blood loss caused by other diseases (e.g., inflammatory bowel disease and chronic kidney disease) [22,36].

The iron deficiency that accompanies other diseases is often more difficult to treat and more severe than iron deficiency alone, which may be one of the reasons why iron deficiency accounts for a high proportion of DALYs in high-SDI region. Poverty, inadequate food diversity and low parental education are important determinants of vitamin A deficiency [37,38,39,40]. In low-SDI region, the above problems are prevalent, making its incidence and DALY rates of vitamin A deficiency highest among all SDI regions. Furthermore, Vitamin A deficiency, rather than protein–energy malnutrition, is the major component of incident cases and DALYs in the low SDI region, suggesting that future policies should not only focus on energy and protein supply but also promote the availability of nutritious foods.

Incidence and DALY rate are important because they help us understand the current negative impact of nutritional deficiencies. However, it is important to explore the temporal trend of them because they can reflect how the disease burden changes, which can further indicate the effectiveness of existing nutrition promotion strategies. In this study, EAPC is an indicator that reflects changes in incidence or DALY rate. The larger its absolute value, the greater decrease (or increase) in the rates. Our results showed that the absolute value of EAPCs increased as SDI decreased.

We speculate that this is because nutritional deficiencies have leveled off in high-SDI region; however, the disease burden in low-SDI region was still high and had more room for decline. This speculation is supported by our findings, as we found that the absolute value of EAPC positively correlated with the incidence and DALY rates in 2010. The low-SDI region is an exception, whose absolute value of EAPC is smaller than that of low-middle SDI region, suggesting that control measures in low-SDI region is relatively less effective.

However, Malawi is a low-SDI country with a marked reduction in disease burden. In this country, *Breastfeeding Promotion* and *Complementary Feeding Promotion* increased exclusive breastfeeding rate, improved child nutrition and reduced child stunting and death. *Purdue Improved Crop Storage and Specific Crop Diversification Strategies* reduced post-harvest losses of vegetables and fruits, avoided single types of food and improved food safety [41]. There are also median and low-median SDI countries whose policies are worth learning from, such as Egypt and Ghana.

Egypt reformed the National Food Subsidy program in 2014. Before the reform, subsidized foods were bread, flour, rice, sugar, edible oil and black tea, mainly for energy supplementation. After the reform, micronutrient-rich foods including lentils, broad beans, meat, chicken, fish, milk and cheese were also added [32]. In Ghana, in addition to *Breastfeeding Promotion*, *Complementary Feeding Promotion* and nutrition-sensitive agriculture, interventions for micronutrients and calcium supplementation during pregnancy were introduced [42].

We believe that in countries and regions with serious nutritional deficiency issues, advocating multi-sectoral collaborative participation, such as promoting nutrition-sensitive agriculture, increasing the availability of nutritious food and strengthening health education, such as promoting breastfeeding and encouraging a balanced diet can better improve children’s nutritional deficiencies.

There are some limitations in our study. First, the data used in this study derived from the GBD database, which only collected the public data and did not include the unpublished data. Second, the data may not be complete in some countries with relatively poor economies. Third, GBD data is only updated to 2019, and thus we cannot obtain data of the last three years. Food safety issues caused by COVID-19 may have impact on nutritional deficiencies [43,44], which we cannot evaluate. The impact of COVID-19 on nutritional deficiency can be further explored in the future.

Furthermore, other factors that may affect nutritional deficiencies, such as breastfeeding, health services, social safety nets, educational settings, agriculture, food systems and food environments are not available in GBD2019. The impact of these factors on global nutritional deficiencies can also be further explored in the future. Finally, we only selected policies implemented in some countries with a large decline in nutritional deficiency cases as a reference.

The effectiveness of a policy can be evaluated using epidemiological methods in the future by combining GBD data. However, our study firstly reported the burden of global, regional and national dietary iron deficiency, iodine deficiency, vitamin A deficiency, protein–energy malnutrition and other nutritional deficiencies in children under 5 years of age from 2010 to 2019, as well as the temporal trend of nutritional deficiencies in the past decades.

These findings help to identify the key regions and countries with serious nutritional deficiency issues among children. In addition, we found that the incidence and DALY rates are negatively associated with the social development status, the low-SDI regions should be the focus of future nutritional deficiencies control. Furthermore, our findings support that dietary quality improvement should be one of the strategies to reduce nutritional deficiency in the future.

## 5. Conclusions

Nutritional deficiencies in children under 5 years of age have been substantially improved globally in the past decades; however, the progress has been unevenly distributed across the globe. The incidence, DALY rate and the EAPC were closely associated with the SDI of a country. Nutritional deficiencies in certain low socioeconomic countries remain worrisome, and policy guarantees and sustained efforts to control nutritional deficiencies are urgently needed.

Our findings support that dietary quality improvement should be one of the measures to reduce nutritional deficiency in the future. We propose that, in countries and regions with serious nutritional deficiency issues, advocating multi-sectoral collaborative participation, such as promoting nutrition-sensitive agriculture, increasing the availability of nutritious food and strengthening health education, such as promoting breastfeeding and encouraging a balanced diet, can better improve children’s nutritional deficiencies.

## Figures and Tables

**Table 1:** The number, rate and temporal trend of incident and DALYs cases among children under 5 years of age, by subcategories, socio-demographic index and regions, 2010–2019.

Characteristics	Incidence					DALYs				
	2010		2019		2010–2019	2010		2019		2010–2019
	No.	IR/10^5^	No.	IR/10^5^	EAPC (%) (95%UI)	No.	DR/10^5^	No.	DR/10^5^	EAPC (%) (95%UI)
**Subcategories of nutritional deficiency**										
**Vitamin A**	144,595,018	22,208.6	100,511,850	15,163.8	−4.4 (−4.6 to −4.1)	710,599	109.1	505,633	76.3	−4.1 (−4.4 to −3.8)
**Iodine**	395,255	60.7	312,948	47.2	−3.1 (−3.5 to −2.6)	18,759	2.9	12,113	1.8	−5.4 (−6.4 to −4.4)
**Dietary iron**	0	0	0	0	0 (0 to 0)	5,424,687	833.2	5,179,895	781.5	−0.7 (−0.7 to −0.7)
**Protein–energy malnutrition**	66,602,727	10,229.6	61,023,138	9206.3	−1.7 (−2.4 to −1.0)	16,518,390	2537.1	9,925,276	1497.4	−5.6 (−5.8 to −5.4)
**Other**	0	0	0	0	0 (0 to 0)	602,624	92.6	381,462	57.5	−5.0 (−5.3 to −4.7)
**Socio-demographic index**										
**Low SDI**	77,499,851	50,331.0	64,648,780	37,838.3	−3.3 (−3.6 to −3.1)	12,654,261	8218.1	9,152,867	5357.1	−4.4 (−4.8 to −3.9)
**Low-middle SDI**	63,374,480	35,451.1	40,781,384	23,669	−4.9 (−5.4 to −4.4)	7,258,770	4060.5	4,421,343	2566.1	−5.1 (−5.2 to −4.9)
**Middle SDI**	35,007,606	19,105.9	23,973,677	13,015.7	−4.7 (−5.1 to −4.2)	2,709,754	1478.9	1,913,652	1038.9	−4.0 (−4.3 to −3.7)
**High-middle SDI**	8,219,025	10,221.1	6,475,620	7836.4	−3.2 (−3.6 to −2.9)	560,604	697.2	437,042	528.9	−3.1 (−3.2 to −3.0)
**High SDI**	2,021,301	3724.6	1,817,801	3466.7	−1.0 (−1.3 to −0.7)	78,265	144.2	69,522	132.6	−1.0 (−1.0 to −0.9)
**Region**										
**Global**	211,593,000	32,498.9	161,847,936	24,417.2	−3.5 (−3.9 to −3.1)	23,275,059	3574.9	16,004,379	2414.5	−4.2 (−4.4 to −4.0)
**Andean Latin America**	744,525	12,984.9	585,325	9239.7	−3.9 (−4.3 to −3.4)	106,016	1849.0	74,227	1171.7	−4.7 (−5.3 to −4.2)
**Australasia**	46,207	2628.2	45,598	2506.5	−0.5 (−0.7 to −0.4)	4318	245.6	4120	226.5	−0.9 (−1.0 to −0.8)
**Caribbean**	664,789	16,818.2	589,157	14,914.6	−1.6 (−1.9 to −1.3)	126,167	3191.8	108,517	2747.1	−1.7 (−2.0 to −1.4)
**Central Asia**	1,333,242	15,680.6	1,200,248	12,538.6	−2.6 (−3.0 to −2.2)	98,809	1162.1	92,950	971.0	−2.0 (−2.1 to −1.8)
**Central Europe**	1,030,090	16,774.7	802,792	14,203.2	−1.9 (−2.0 to −1.8)	27,267	444.0	22,435	396.9	−1.1 (−1.2 to −1.1)
**Central Latin America**	3,563,427	15,438.1	2,592,401	11,970.6	−3.1 (−3.4 to −2.7)	324,765	1407.0	212,374	980.7	−3.9 (−4.3 to −3.6)
**Central Sub-Saharan Africa**	13,318,287	71,995.4	10,737,695	51,872.0	−3.8 (−4.1 to −3.6)	1,684,209	9104.4	820,961	3965.9	−9.2 (−9.6 to −8.7)
**East Asia**	8,087,107	11,137.3	7,265,853	8635.6	−2.9 (−3.3 to −2.5)	273,307	376.4	197,281	234.5	−4.8 (−5.2 to −4.5)
**Eastern Europe**	784,037	6577.9	763,059	6185.4	−1.1 (−1.6 to −0.6)	33,719	282.9	29,051	235.5	−2.1 (−2.2 to −2.0)
**Eastern Sub-Saharan Africa**	30,470,720	53,385.8	26,568,159	41,423.1	−2.8 (−3.0 to −2.7)	6,008,221	10,526.6	3,584,055	5588.0	−6.7 (−7.4 to −6.0)
**High-income Asia Pacific**	380,629	4738.8	319,033	4378.4	−1.0 (−1.3 to −0.7)	17,260	214.9	14,375	197.3	−0.9 (−1.1 to −0.8)
**High-income North America**	539,065	2414.9	453,379	2160.6	−1.4 (−1.7 to −1.1)	13,619	61.0	13,813	65.8	0.9 (0.8 to 1.0)
**North Africa and Middle East**	15,029,443	25,188.1	12,214,970	20,453.8	−2.6 (−3.0 to −2.1)	1,018,213	1706.4	755,452	1265.0	−3.3 (−3.3 to −3.2)
**Oceania**	583,853	38,304.1	575,019	31,070.1	−2.5 (−2.9 to −2.1)	37,750	2476.6	37,476	2025.0	−2.2 (−2.5 to −1.9)
**South Asia**	80,495,955	45,888.8	53,510,848	32,548.3	−4.3 (−4.9 to −3.7)	7,805,120	4449.5	4,804,447	2922.3	−4.7 (−4.9 to −4.5)
**Southeast Asia**	21,278,763	36,034.4	13,792,558	25,314.5	−4.2 (−4.7 to −3.6)	887,738	1503.3	606,269	1112.7	−3.2 (−3.3 to −3.1)
**Southern Latin America**	769,282	15,784.7	612,018	12,607.6	−2.6 (−2.8 to −2.3)	30,155	618.8	25,864	532.8	−1.6 (−2.2 to −1.1)
**Southern Sub-Saharan Africa**	2,293,235	28,544.1	1,825,978	22,553.1	−2.8 (−3.1 to −2.5)	345,760	4303.7	331,076	4089.2	−1.0 (−1.8 to −0.1)
**Tropical Latin America**	3,130,047	18,153.3	2,310,875	14,324.3	−2.7 (−3.2 to −2.2)	290,412	1684.3	173,953	1078.3	−5.0 (−5.7 to −4.2)
**Western Europe**	970,260	4242.0	869,238	3951.5	−0.9 (−1.1 to −0.6)	27,749	121.3	24,773	112.6	−0.9 (−1.0 to −0.7)
**Western Sub-Saharan Africa**	26,080,037	41,552.9	24,213,730	33,291.8	−2.6 (−2.9 to −2.2)	4,114,484	6555.5	4,070,910	5597.1	−1.0 (−1.7 to −0.3)

## Data Availability

The data in this article is available on the GBD 2019 database at http://ghdx.healthdata.org/gbd-results-tool (accessed on 27 March 2022).

## Supplementary Materials

The following supporting information can be downloaded at: https://www.mdpi.com/article/10.3390/nu14132685/s1, Figure S1: Change in the number of nutritional deficiencies in children under 5 years of age in 2019 compared to 2010 (IR: Incidence rate; DR: DALYs rate; EAPC: Estimate annual percentage change; C.A.R.: Central African Republic; Dem. Rep. of Congo: Democratic Republic of the Congo; B. Faso: Burkina Faso; and Syr.: Syrian Arab Republic); Figure S2: Global burden of nutritional deficiencies in children under 5 years of age; Figure S3: Contribution of dietary iron deficiency, iodine deficiency, vitamin A deficiency, protein–energy malnutrition and other cause to nutritional deficiency cases and DALYs, globally and by region, in 2010 and 2019; Figure S4: Cluster of countries and territories with similar EAPC of incidence and DALYs of nutritional deficiencies in children under 5 years of age; Figure S5: Cluster of countries and territories with similar incidence and DALYs of nutritional deficiencies in children under 5 years of age in 2019; Figure S6: Cluster of countries and territories with similar incidence and DALYs of nutritional deficiencies in children under 5 years of age in 2010; Table S1: Incidence and temporal trend of five nutritional deficiencies subcategories among children under 5 years of age in five SDI regions and 21 GBD regions from 2010 to 2019; Table S2. DALY rate and temporal trend of five nutritional deficiencies subcategories among children under 5 years of age in five SDI regions and 21 GBD regions from 2010 to 2019.
