# Supplementary material for: Global Burden of Nutritional Deficiencies among Children under 5 Years of Age from 2010 to 2019
Source: Nutrients. 2022 Jun 28;14(13):2685. doi: 10.3390/nu14132685 (PMC9268233; doi:10.3390/nu14132685)
Supplement: Supplementary file 1 [file nutrients-14-02685-s001.zip › nutrients-1770786-supplementary.pdf]

Global Burden of Nutritional Deficiencies among Children Under 5 Years of Age from 2010 to 2019

Figure S1. Change in the number of nutritional deficiencies in children under 5 years of age in 2019 compared to 2010. (A) Change in incidence cases of nutritional deficiencies in children under 5 years of age compared to 2010; (B) Change in DALYS cases of nutritional deficiencies in children under 5 years of age compared to 2010. (IR: Incidence rate; DR: DALYs rate; EAPC: Estimate annual percentage change; C.A.R.: Central African Republic; Dem. Rep. of Congo: Democratic Republic of the Congo; B. Faso: Burkina Faso; Syr.: Syrian Arab Republic)

Figure S2. Global burden of nutritional deficiencies in children under 5 years of age. (a) EPAC of the incidence rate of global nutritional deficiencies in children under 5 years of age from 2010 to 2019. (b) EPAC of the DALYs rate of global nutritional deficiencies in children under 5 years of age from 2010 to 2019. (IR: Incidence rate; DR: DALYS rate; EAPC: Estimate annual percentage change)

Figure S3. Contribution of dietary iron deficiency, iodine deficiency, vitamin A deficiency, protein-energy malnutrition and other cause to nutritional deficiency cases and DALYs, globally and by region, in 2010 and 2019.

Figure S4. Cluster of countries and territories with similar EAPC of incidence and DALY rate of nutritional deficiencies in children under 5 years of age.

Figure S5. Cluster of countries and territories with similar incidence and DALY rate of nutritional deficiencies in children under 5 years of age in 2019.

Figure S6. Cluster of countries and territories with similar incidence and DALY rate of nutritional deficiencies in children under 5 years of age in 2010.

Table S1. Incidence and temporal trend of 5 nutritional deficiencies subcategories among children under 5 years of age in 5 SDI regions and 21 GBD regions from 2010 to 2019

Table S2. DALY rate and temporal trend of 5 nutritional deficiencies subcategories among children under 5 years of age in 5 SDI regions and 21 GBD regions from 2010 to 2019

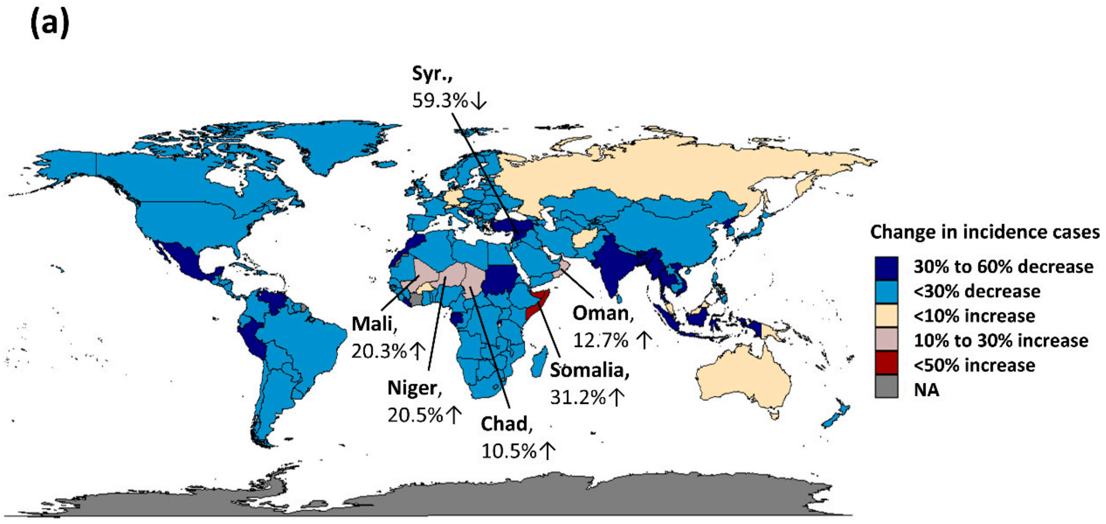

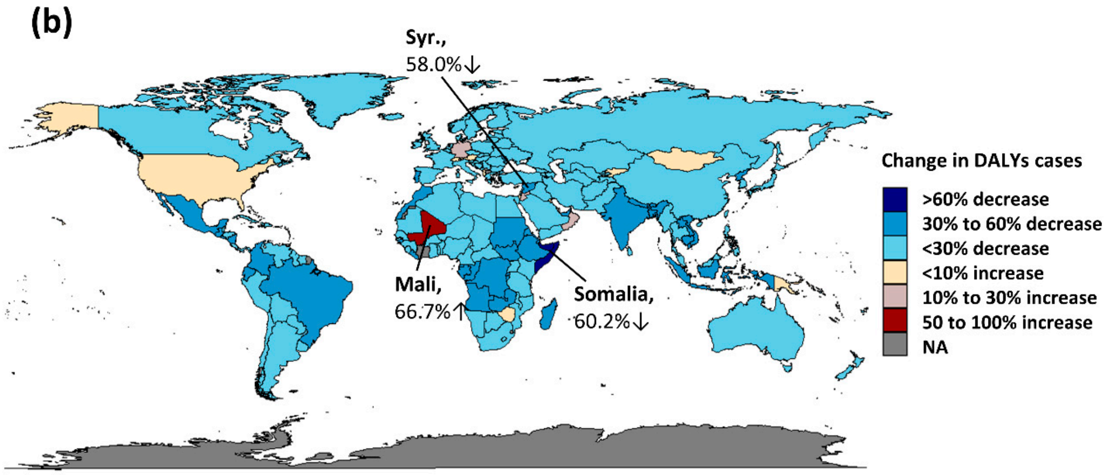

Figure S1. Change in the number of nutritional deficiencies in children under 5 years of age in 2019 compared to 2010. (a) Change in incidence cases of nutritional deficiencies in children under 5 years of age compared to 2010; (b) Change in DALYS cases of nutritional deficiencies in children under 5 years of age compared to 2010. (IR: Incidence rate; DR: DALYs rate; EAPC: Estimate annual percentage change; C.A.R.: Central African Republic; Dem. Rep. of Congo: Democratic Republic of the Congo; B. Faso: Burkina Faso； Syr.: Syrian Arab Republic)

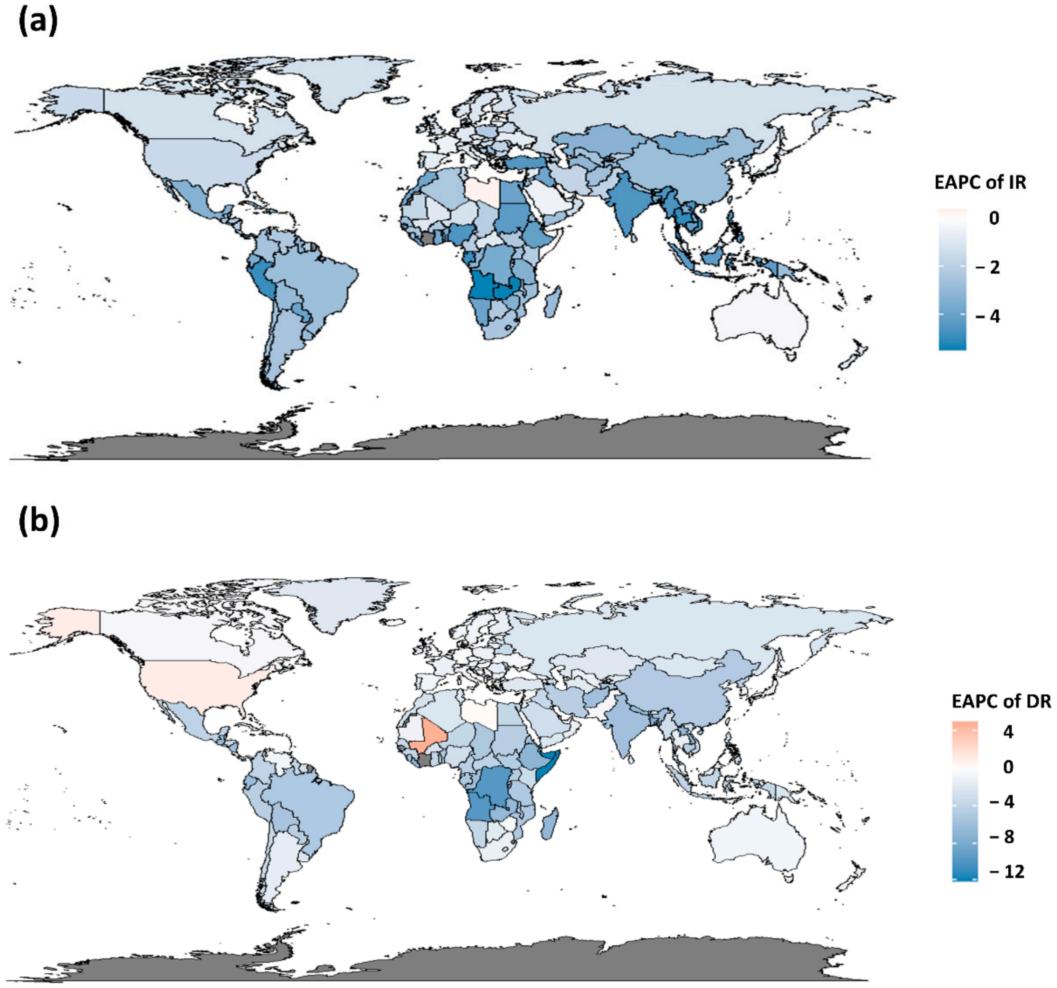

Figure S2. Global burden of nutritional deficiencies in children under 5 years of age. (a) EPAC of the incidence rate of global nutritional deficiencies in children under 5 years of age from 2010 to 2019. (b) EPAC of the DALYs rate of global nutritional deficiencies in children under 5 years of age from 2010 to 2019. (IR: Incidence rate; DR: DALYS rate; EAPC: Estimate annual percentage change)

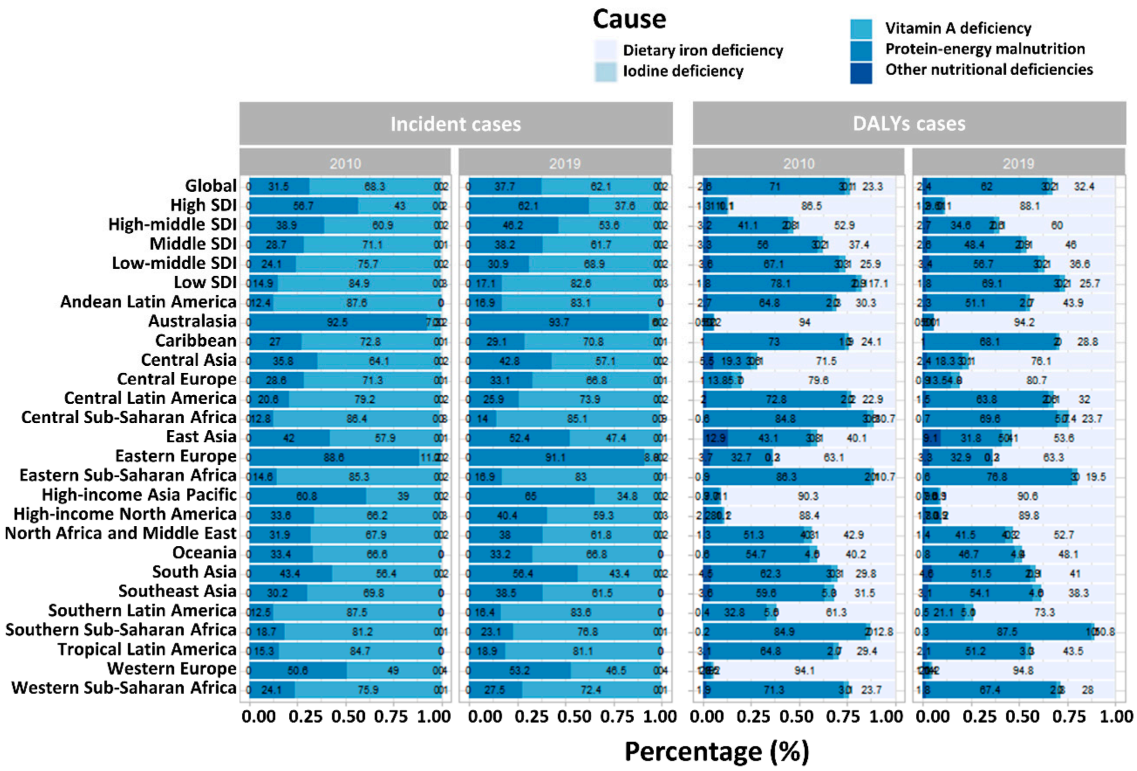

Figure S3. Contribution of dietary iron deficiency, iodine deficiency, vitamin A deficiency, protein-energy malnutrition and other cause to nutritional deficiency cases and DALYs, globally and by region, in 2010 and 2019.

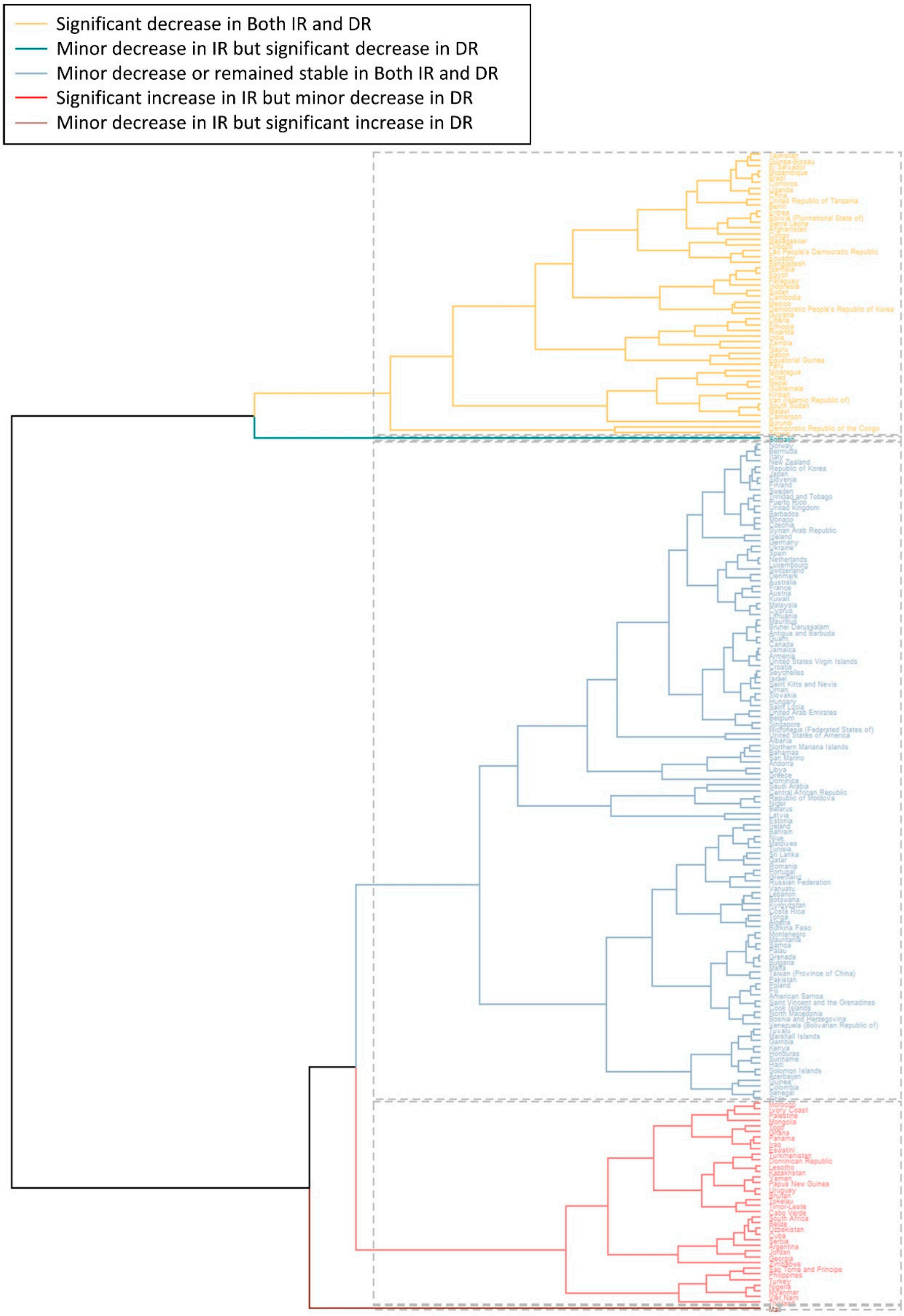

Figure S4. Cluster of countries and territories with similar EAPC of incidence and DALY rate of nutritional deficiencies in children under 5 years of age.

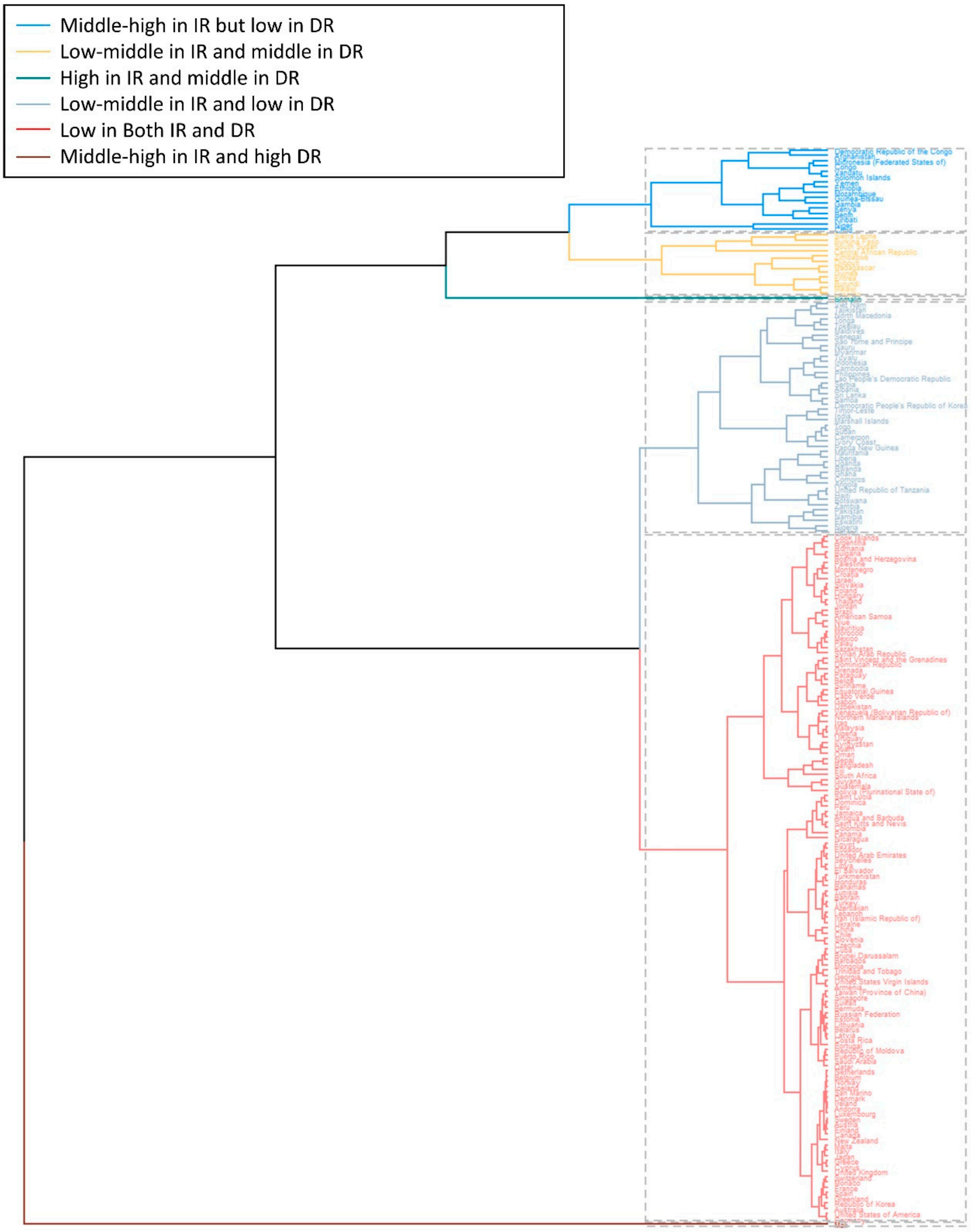

Figure S5. Cluster of countries and territories with similar incidence and DALY rate of nutritional deficiencies in children under 5 years of age in 2019.

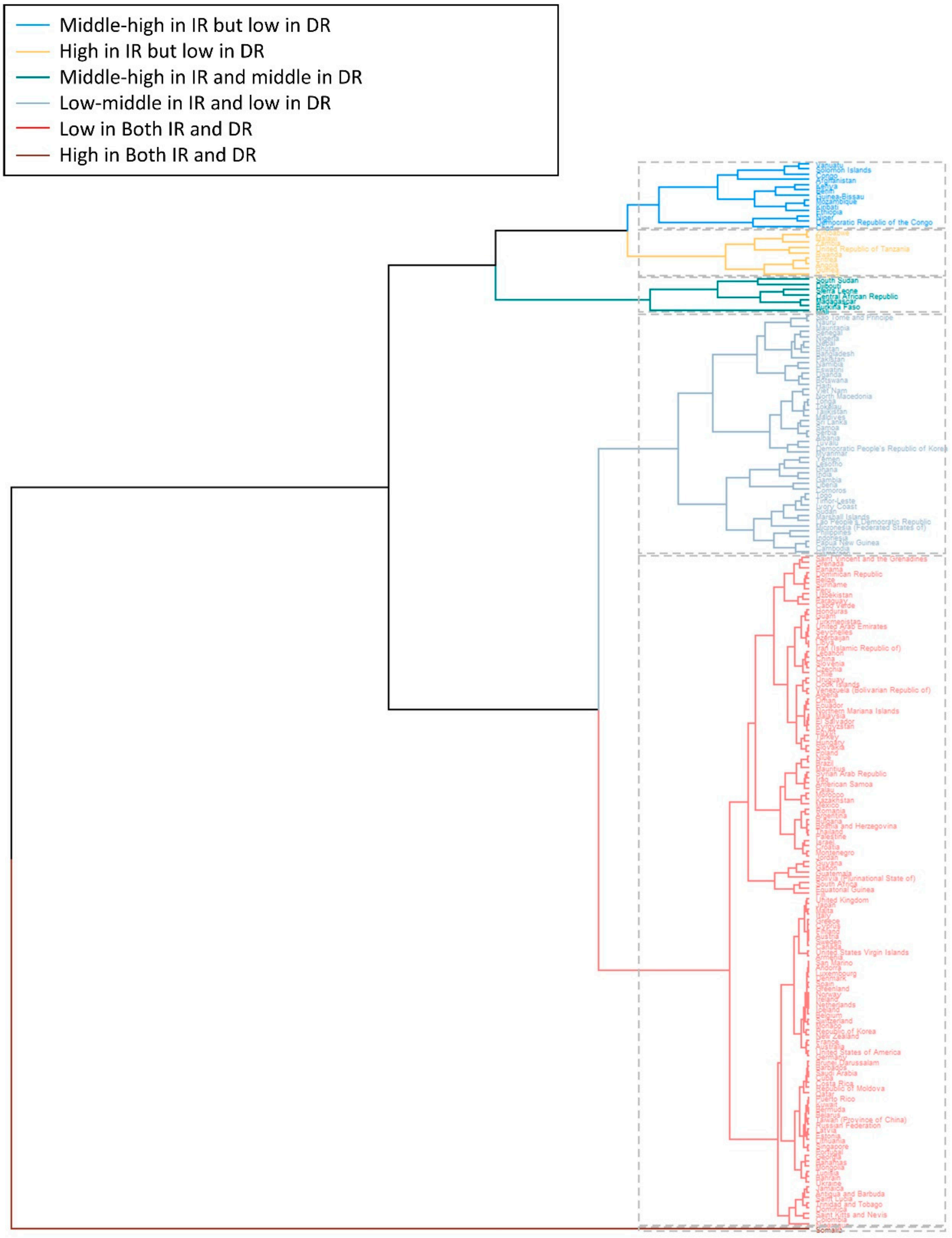

Figure S6. Cluster of countries and territories with similar incidence and DALY rate of nutritional deficiencies in children under 5 years of age in 2010.

Table S1. Incidence and temporal trend of 5 nutritional deficiencies subcategories among children under 5 years of age in 5 SDI regions and 21 GBD regions from 2010 to 2019.

Region	Vitamin A deficiency		Iodine deficiency		Dietary iron deficiency		Protein-energy malnutrition		Other	
	2019 IR/10^5	2010 - 2019 EAPC (%) (95%UI)	2019 IR/10^5	2010 - 2019 EAPC (%) (95%UI)	2019 IR/10^5	2010-2019 EAPC (%) (95%UI)	2019 IR/10^5	2010-2019 EAPC (%)	2019 IR/10^5	2010-2019 EAPC (%) (95%UI)
**Global**	15,163.8	-4.4 (-4.6 to -4.1)	47.2	-3.1 (-3.5 to -2.6)	0	0 (0 to 0)	9206.3	-1.7 (-2.4 to -1)	0	0 (0 to 0)
**High-middle SDI**	4201.8	-4.3 (-4.4 to -4.2)	16.6	-1.3 (-1.7 to -1)	0	0 (0 to 0)	3617.9	-1.8 (-2.5 to -1)	0	0 (0 to 0)
**High SDI**	1305.0	-2.3 (-2.4 to -2.2)	8.4	-0.3 (-0.3 to -0.2)	0	0 (0 to 0)	2153.2	-0.2 (-0.6 to 0.3)	0	0 (0 to 0)
**Low-middle SDI**	16,307.4	-5.7 (-6.0 to -5.4)	51.1	-2.8 (-3.4 to -2.2)	0	0 (0 to 0)	7310.5	-2.8 (-3.9 to -1.7)	0	0 (0 to 0)
**Low SDI**	31,257.5	-3.6 (-3.7 to -3.4)	97.1	-4.2 (-4.6 to -3.7)	0	0 (0 to 0)	6483.7	-2.1 (-2.7 to -1.5)	0	0 (0 to 0)
**Middle SDI**	8026.2	-5.9 (-6.1 to -5.7)	22.1	-1.8 (-2.3 to -1.4)	0	0 (0 to 0)	4967.4	-2.1 (-3.2 to -1.1)	0	0 (0 to 0)
**Andean Latin America**	7676.7	-4.3 (-4.6 to -3.9)	2.1	-0.9 (-1.0 to -0.8)	0	0 (0 to 0)	1560.9	-1.3 (-2.4 to -0.1)	0	0 (0 to 0)
**Australasia**	150.7	-2.7 (-3.0 to -2.4)	6.0	-0.3 (-0.3 to -0.2)	0	0 (0 to 0)	2349.7	-0.4 (-0.5 to -0.2)	0	0 (0 to 0)
**Caribbean**	10,557.2	-1.8 (-2.0 to -1.6)	21.7	-0.4 (-0.7 to -0.2)	0	0 (0 to 0)	4335.7	-1.1 (-2.3 to 0.1)	0	0 (0 to 0)
**Central Asia**	7153.3	-3.7 (-3.9 to -3.6)	20.1	-2.2 (-2.6 to -1.9)	0	0 (0 to 0)	5365.2	-0.8 (-1.5 to -0.1)	0	0 (0 to 0)
**Central Europe**	9487.9	-2.6 (-2.6 to -2.5)	8.7	-1.0 (-1.1 to -0.8)	0	0 (0 to 0)	4706.6	-0.3 (-0.7 to 0.2)	0	0 (0 to 0)
**Central Latin America**	8847.9	-3.7 (-3.8 to -3.5)	28.0	0.1 (0 to 0.1)	0	0 (0 to 0)	3094.7	-1.0 (-2.3 to 0.2)	0	0 (0 to 0)
**Central Sub-Saharan Africa**	44,156.8	-4.0 (-4.2 to -3.8)	455.6	-3.3 (-3.7 to -3)	0	0 (0 to 0)	7259.6	-3.0 (-3.7 to -2.2)	0	0 (0 to 0)
**East Asia**	4095.9	-4.6 (-5.1 to -4.2)	11.4	0.7 (0.5 to 1)	0	0 (0 to 0)	4528.2	-1 (-1.8 to -0.2)	0	0 (0 to 0)
**Eastern Europe**	541.8	-3.3 (-3.5 to -3.2)	10.8	-0.6 (-0.9 to -0.3)	0	0 (0 to 0)	5632.8	-0.9 (-1.4 to -0.3)	0	0 (0 to 0)
**Eastern Sub-Saharan Africa**	34,384.4	-3.1 (-3.2 to -3.1)	57.6	-6.8 (-7.5 to -6)	0	0 (0 to 0)	6981.1	-1.3 (-1.8 to -0.9)	0	0 (0 to 0)
**High-income Asia Pacific**	1525.1	-2.1 (-2.2 to -2.1)	7.4	-0.3 (-0.5 to -0.2)	0	0 (0 to 0)	2845.9	-0.4 (-0.9 to 0.1)	0	0 (0 to 0)

**High-income North America**	1280.7	-2.5 (-2.7 to -2.2)	6.6	-0.1 (-0.2 to 0.1)	0	0 (0 to 0)	873.3	0.4 (0 to 0.8)	0	0 (0 to 0)
**North Africa and Middle East**	12,635.8	-3.5 (-3.8 to -3.1)	42.7	-0.9 (-1.6 to -0.1)	0	0 (0 to 0)	7775.2	-0.8 (-1.4 to -0.1)	0	0 (0 to 0)
**Oceania**	20,762.6	-2.4 (-2.5 to -2.3)	1.4	-2.9 (-3.5 to -2.3)	0	0 (0 to 0)	10,306.1	-2.8 (-4 to -1.6)	0	0 (0 to 0)
**South Asia**	14,138.7	-6.9 (-7.3 to -6.5)	64.2	-3.2 (-4 to -2.5)	0	0 (0 to 0)	18,345.5	-1.6 (-2.2 to -0.9)	0	0 (0 to 0)
**Southeast Asia**	15,573.4	-5.3 (-5.6 to -5.1)	5.5	-3 (-3.4 to -2.5)	0	0 (0 to 0)	9735.6	-1.8 (-3 to -0.6)	0	0 (0 to 0)
**Southern Latin America**	10,539.4	-3.0 (-3.1 to -2.9)	5.5	-0.4 (-0.6 to -0.2)	0	0 (0 to 0)	2062.7	0.1 (-0.6 to 0.9)	0	0 (0 to 0)
**Southern Sub-Saharan Africa**	17,324.9	-3.4 (-3.6 to -3.2)	26.6	-0.5 (-0.6 to -0.4)	0	0 (0 to 0)	5201.6	-0.6 (-1.5 to 0.3)	0	0 (0 to 0)
**Tropical Latin America**	11,612.4	-3.1 (-3.3 to -2.8)	3.1	-0.1 (-0.2 to 0.1)	0	0 (0 to 0)	2708.7	-1.1 (-2.9 to 0.6)	0	0 (0 to 0)
**Western Europe**	1836.2	-1.4 (-1.4 to -1.3)	14.7	-0.7 (-0.7 to -0.6)	0	0 (0 to 0)	2100.5	-0.4 (-0.9 to 0)	0	0 (0 to 0)
**Western Sub-Saharan Africa**	24,101.8	-3 (-3.2 to -2.8)	26.2	-1.0 (-1.1 to -1)	0	0 (0 to 0)	9163.8	-1.3 (-1.9 to -0.7)	0	0 (0 to 0)

Table S2. DALY rate and temporal trend of 5 nutritional deficiencies subcategories among children under 5 years of age in 5 SDI regions and 21 GBD regions from 2010 to 2019.

Region	Vitamin A deficiency		Iodine deficiency		Dietary iron deficiency		Protein-energy malnutrition		Other nutritional deficiencies	
	2019	2010 - 2019	2019	2010 - 2019	2019	2010-2019	2019	2010-2019	2019	2010-2019
	DR/10^5	EAPC (%) (95%UI)	DR/10^5	EAPC (%) (95%UI)	DR/10^5	EAPC (%) (95%UI)	DR/10^5	EAPC (%) (95%UI)	DR/10^5	EAPC (%) (95%UI)
**Global**	76.3	-4.1 (-4.4 to -3.8)	1.8	-5.4 (-6.4 to -4.4)	781.5	-0.7 (-0.7 to -0.7)	1497.4	-5.6 (-5.8 to -5.4)	57.5	-5 (-5.3 to -4.7)
**Low SDI**	173.2	-3.6 (-3.8 to -3.4)	4.0	-5.8 (-6.8 to -4.9)	1377.9	-0.2 (-0.2 to -0.1)	3703.0	-5.6 (-6.1 to -5)	99.0	-4.2 (-4.7 to -3.7)
**Low-middle SDI**	82.2	-5.6 (-5.9 to -5.3)	2.1	-5.8 (-6.6 to -4.9)	939.0	-1.2 (-1.3 to -1.2)	1456.2	-6.9 (-7.2 to -6.6)	86.5	-5.6 (-5.9 to -5.2)
**Middle SDI**	30.1	-5.2 (-5.6 to -4.8)	0.6	-5.6 (-7.1 to -4.1)	478.4	-1.6 (-1.7 to -1.5)	502.4	-5.6 (-6.1 to -5.2)	27.4	-6.4 (-6.7 to -6.1)
**High-middle SDI**	14.0	-3.7 (-4.1 to -3.4)	0.4	-1.5 (-1.8 to -1.2)	317.2	-1.7 (-1.7 to -1.6)	183.0	-5.0 (-5.4 to -4.7)	14.3	-4.7 (-5.1 to -4.2)
**High SDI**	1.3	-2.2 (-2.3 to -2.1)	0.2	-0.3 (-0.4 to -0.2)	116.8	-0.8 (-0.8 to -0.7)	12.7	-2.4 (-2.6 to -2.2)	1.6	-1.6 (-1.9 to -1.2)
**Andean Latin America**	31.7	-3.3 (-3.5 to -3.0)	0.01	-0.8 (-0.9 to -0.7)	514.2	-1 (-1.1 to -0.9)	598.4	-7.1 (-7.8 to -6.4)	27.3	-6.0 (-6.8 to -5.2)
**Australasia**	0.3	-3.6 (-4 to -3.2)	0.1	0.2 (0 to 0.3)	213.3	-0.9 (-1 to -0.7)	11.4	-1 (-1.2 to -0.7)	1.3	-1.6 (-2.0 to -1.2)
**Caribbean**	56.0	-0.8 (-1.1 to -0.5)	0.8	-0.6 (-0.8 to -0.3)	791.6	0.4 (0.3 to 0.6)	1871.9	-2.5 (-2.8 to -2.1)	26.8	-2.3 (-2.9 to -1.7)
**Central Asia**	30.5	-3.5 (-3.7 to -3.4)	0.6	-2.8 (-3.3 to -2.3)	739.0	-1.3 (-1.3 to -1.3)	177.4	-2.6 (-2.8 to -2.3)	23.5	-11.2 (-13.1 to -9.2)
**Central Europe**	18.9	-3.0 (-3.1 to -2.9)	0.2	-1.0 (-1.2 to -0.9)	320.4	-1.0 (-1.1 to -0.9)	53.7	-1.2 (-1.5 to -1)	3.8	-1.3 (-2.0 to -0.6)
**Central Latin America**	25.7	-2.3 (-2.4 to -2.2)	0.7	4.8 (3.4 to 6.3)	313.9	-0.2 (-0.4 to -0.1)	625.7	-5.4 (-5.9 to -4.9)	14.7	-7.3 (-7.7 to -6.9)
**Central Sub-Saharan Africa**	225.4	-4.4 (-4.7 to -4.2)	15.7	-5.8 (-6.9 to -4.6)	938.8	-0.6 (-0.8 to -0.3)	2759.0	-11.2 (-11.8 to -10.5)	27.0	-7.3 (-7.7 to -7)
**East Asia**	12.6	-1.9 (-2.6 to -1.3)	0.2	0.7 (0.4 to 1)	125.7	-1.8 (-2.1 to -1.5)	74.6	-7.8 (-8.4 to -7.3)	21.4	-8.3 (-8.9 to -7.8)
**Eastern Europe**	0.6	-4.7 (-5 to -4.4)	0.4	-0.6 (-0.9 to -0.3)	149.1	-2.0 (-2.2 to -1.9)	77.5	-2.1 (-2.4 to -1.7)	7.8	-3.2 (-3.4 to -2.9)
**Eastern Sub-Saharan Africa**	168.8	-3.1 (-3.2 to -3.0)	2.4	-8.0 (-9.1 to -6.9)	1092.1	-0.3 (-0.4 to -0.3)	4289.1	-7.9 (-8.7 to -7.1)	35.6	-9.3 (-11.9 to -6.7)

**High-income Asia Pacific**	1.7	-2.9 (-3.0 to -2.8)	0.1	-0.3 (-0.5 to -0.1)	178.8	-0.9 (-1 to -0.8)	14.9	-1.2 (-1.2 to -1.2)	1.8	-1.3 (-1.5 to -1.1)
**High-income North America**	0.6	-1.8 (-2 to -1.6)	0.1	-0.1 (-0.3 to 0.1)	59.1	1.0 (0.9 to 1.1)	4.8	0.2 (-0.5 to 0.8)	1.2	-0.9 (-1.2 to -0.7)
**North Africa and Middle East**	54.1	-3.6 (-4 to -3.3)	2.1	-1.0 (-2.0 to 0)	666.5	-1.0 (-1.2 to -0.9)	524.7	-5.5 (-5.7 to -5.3)	17.6	-2.7 (-3.2 to -2.2)
**Oceania**	89.7	-2.4 (-2.5 to -2.3)	0.1	-5.3 (-6.3 to -4.3)	973.3	-0.3 (-0.4 to -0.2)	946.5	-3.9 (-4.4 to -3.4)	15.4	0.3 (0.1 to 0.5)
**South Asia**	84.0	-6.3 (-6.7 to -5.9)	2.9	-6.2 (-7.3 to -5.1)	1197.9	-1.1 (-1.1 to -1.1)	1504.5	-6.8 (-7.2 to -6.4)	133.1	-4.4 (-4.9 to -3.9)
**Southeast Asia**	50.8	-4.9 (-5.3 to -4.6)	0.2	4.3 (1.9 to 6.8)	426	-1.1 (-1.3 to -0.9)	601.5	-4.2 (-4.4 to -4)	34.3	-4.9 (-5 to -4.8)
**Southern Latin America**	27.2	-2.6 (-2.7 to -2.4)	0.1	-0.5 (-0.8 to -0.2)	390.7	0.3 (0.2 to 0.4)	112.2	-6.5 (-8.2 to -4.8)	2.5	-0.5 (-0.8 to -0.2)
**Southern Sub-Saharan Africa**	59.7	-4.4 (-4.8 to -4.1)	0.5	-0.9 (-1 to -0.8)	441.1	-2.5 (-2.8 to -2.2)	3576.7	-0.7 (-1.8 to 0.4)	11.2	0.4 (-0.2 to 1)
**Tropical Latin America**	35.3	-2.7 (-2.9 to -2.5)	0.1	0 (-0.2 to 0.1)	468.5	-0.6 (-0.6 to -0.6)	551.7	-7.7 (-8.8 to -6.6)	22.7	-8.6 (-10 to -7.1)
**Western Europe**	1.6	-2 (-2.2 to -1.9)	0.3	-0.7 (-0.8 to -0.6)	106.8	-0.8 (-0.9 to -0.7)	2.7	-2.5 (-2.9 to -2.1)	1.3	-1.7 (-2.1 to -1.3)
**Western Sub-Saharan Africa**	157.0	-2.9 (-3.1 to -2.6)	0.8	-2.9 (-3.5 to -2.3)	1567	0.2 (0.1 to 0.3)	3773.4	-1.3 (-2.3 to -0.4)	99.0	-2.1 (-2.7 to -1.5)
